# Probiotic *Lacticaseibacillus casei* 2S-1 Attenuates *Escherichia coli*-Induced Enteritis via Gut Microbiota Modulation and Host Gene Regulation

**DOI:** 10.3390/cells15171551

**Published:** 2026-08-27

**Authors:** Yingchao Li, Mingshuai Chen, Chenyang Shi, Qirui Zang, Yaolong Song, Wanjing Jin, Qian Ma, Binhuan Ma, Panpan Tong, Zhanqiang Su, Yi Zhang, Shicheng Wan, Aili Aierken, Mengfei Zhang

**Affiliations:** 1 College of Veterinary Medicine, Xinjiang Agricultural University, Urumqi 830052, China; liyingchao0409@163.com (Y.L.); chenms416@outlook.com (M.C.); 17634384908@163.com (C.S.); 19199130252@163.com (Q.Z.); 17726785228@163.com (Y.S.); 13319912981@163.com (W.J.); 15339306406@163.com (Q.M.); 15750679553@163.com (B.M.); tongpanpan@xjau.edu.cn (P.T.); szq00009@163.com (Z.S.); zydy052@163.com (Y.Z.); 2 Urumqi Field Scientific Observation and Research Station for Animal Diseases, Ministry of Agriculture and Rural Affairs, Urumqi 830052, China; 3 Xinjiang Key Laboratory of New Drug Research and Development for Herbivorous Animals, Urumqi 830052, China; 4 Xinjiang Regional Key Laboratory of Clinical Veterinary Medicine Research, Urumqi 830052, China; 5 Herbivorous Animal Biomedicine and Diagnostic Technology Engineering Research Center, Xinjiang Uygur Autonomous Region, Urumqi 830052, China; 6 College of Veterinary Medicine, Northwest A&F University, Yangling 712100, China; shicheng_wan@nwafu.edu.cn; 7 State Key Laboratory of Pathogenesis, Prevention and Treatment of High Incidence Diseases in Central Asia, Xinjiang Medical University, Urumqi 830011, China; alijan@xjmu.edu.cn

**Keywords:** *Lacticaseibacillus casei*, *Escherichia coli*, gut microbiota, gene regulation, oxidative stress

## Abstract

Maintaining gut microbial homeostasis is crucial for host health, whereas infection with *Escherichia coli* (*E. coli*) is a major contributor to intestinal inflammation and microbial dysbiosis. Recent research has focused on probiotic strategies for managing enteric inflammatory disorders. Previous studies have shown that beneficial microorganisms show protection through modulating host immune responses, enhancing intestinal epithelial barrier integrity, and inhibiting pathogenic bacteria. To evaluate the prophylactic effectiveness of a recently isolated strain, *Lacticaseibacillus casei* 2S-1, in a murine model of *E. coli*-induced enteritis, this study focuses on interactions within the microbiota–intestinal–immune axis, together with host transcriptional responses and pathway enrichment associated with oxidative stress and mitochondrial function. In vitro analysis of probiotic features, including growth dynamics, acidogenic capacity, and tolerance to acidic and bile salt environments, as well as genetic safety profiling, followed the methodical isolation and taxonomic identification of *L. casei* 2S-1. A preventive intervention protocol was established, and a murine model of enteritis was induced by exposure to *E. coli*. Histopathological analyses were performed to observe in vivo safety and protective efficacy. Changes in gut microbial structure were characterized by *16S* rRNA gene sequencing, while host responses were identified by intestinal immunohistochemistry and transcriptome profiling. *L. casei* 2S-1 showed probiotic properties. In vitro analyses showed that the strain exhibited tolerance to acidic and bile salt conditions, and its untreated culture supernatant showed antimicrobial activity against pathogenic bacteria. Its safety profile was supported by genomic analysis, which verified the lack of virulence-associated genes and antibiotic resistance factors. In vivo, *L. casei* 2S-1 pretreatment reduced mortality and intestinal inflammation, modulated gut microbial composition, and preserved intestinal barrier-associated protein expression in infected mice. This study provides experimental evidence supporting the prophylactic effects of *L. casei* 2S-1 and its associations with gut microbiota modulation and host transcriptional responses, providing a foundation for further investigation of probiotic-based preventive strategies against intestinal infections.

## 1. Introduction

*Escherichia coli* (*E. coli*) is a common, opportunistic, Gram-negative bacterium found in both human and animal gastrointestinal microbiomes. A major cause of infectious diarrhea worldwide, pathogenic strains of this organism harbor a wide range of virulence factors that trigger inflammatory cascades in the host intestinal tract, resulting in clinical manifestations such as fever, vomiting, and diarrhea [1]. According to epidemiological data, *E. coli*’s increasing virulence and cross-species transmissibility pose a public health risk and raise morbidity and mortality rates [2]. In livestock production systems, enteritis caused by this pathogen in ruminants is characterized by persistent diarrhea, resulting in significant impairment of growth performance and survival, particularly in neonatal animals. Current clinical management predominantly relies on antibiotic administration; however, extensive antimicrobial use has precipitated a complex, escalating crisis. The urgency of this problem is further highlighted by the World Health Organization (WHO), which has identified antimicrobial-resistant Enterobacterales, including resistant *Escherichia coli*, as priority bacterial pathogens requiring intensified surveillance, research, and the development of new antimicrobial strategies [3]. This gap necessitates exploring alternative therapeutic strategies. Advances in microbiome science have promoted the development of microecological interventions that precisely modulate host–microbiota interactions, providing promising approaches for controlling infectious enteritis in animal production systems.

The global rise in antimicrobial resistance has significantly reduced the efficacy of conventional treatments for infections caused by multidrug-resistant “superbugs,” highlighting the urgent need for antibiotic alternatives. Among emerging strategies, probiotic-based interventions have received greater attention for their ability to modulate the intestinal microenvironment selectively. Numerous studies demonstrate that beneficial microorganisms produce antimicrobial peptides and compete with pathogenic bacteria for ecological niches, thereby exerting probiotic effects. They also improve the integrity of the intestinal barrier by upregulating tight junction proteins, regulate T-cell responses, and restore the balance of pro- and anti-inflammatory cytokines [4,5].

The functional properties of probiotic strains are closely associated with their ecological origin and evolutionary adaptation. The gastrointestinal tract of Xinjiang cattle, adapted to extreme environmental conditions such as high altitude, low temperature, and aridity, harbors a microbiota with enhanced stress tolerance. This microbial community may be enriched for traits such as bile salt resistance, tolerance to extreme pH, and enzymatic systems that facilitate the degradation of plant fiber [6]. These characteristics may confer distinct advantages for the development and application of probiotics in host health management. Among established probiotic taxa, Lactobacillus and Bifidobacterium species are important to conventional formulations. *Lacticaseibacillus casei* has emerged as a promising probiotic candidate, capable of producing exopolysaccharides [7] and modulating the T- helper (Th1/Th2) immune balance [8,9].

In this study, a bovine-derived *Lacticaseibacillus casei* strain (*L. casei* 2S-1) was successfully isolated and taxonomically identified, showing considerable probiotic potential. A comprehensive in vitro safety analysis, along with validation in an in vivo model, confirmed its favorable host adaptability. Furthermore, a mouse model of *E. coli* enteritis was used to assess its preventive efficacy.

## 2. Materials and Methods

### 2.1. Isolation and Identification of Bovine-Derived L. casei

Rumen content samples were collected aseptically through the rumen fistulae of fistulated dairy cows and immediately transferred into sterile anaerobic sampling containers for subsequent bacterial isolation. The samples were serially diluted to 10^−3^ using sterile saline in 10-fold increments. MRS broth, MRS agar, and CaCO_3_ were purchased from Qingdao Hope Bio-Technology Co., Ltd. (Qingdao, China). Aliquots of approximately 100 μL were evenly distributed onto CaCO_3_-enriched MRS agar and incubated for 24 h at 37 °C under anaerobic conditions. After being chosen and grown in MRS broth, colonies exhibiting characteristic calcium-dissolution halos were purified using serial streak plating. Purified isolates were subjected to biochemical tests after staining to verify their Gram-positive status [10]. The isolate was initially identified by 16S rRNA gene sequencing and further characterized by whole-genome sequencing. To achieve robust species-level identification, average nucleotide identity (ANI) analysis was performed using FastANI v1.34. The draft genome assembly of strain 2S-1 was compared with type-strain genomes of closely related members of the *Lacticaseibacillus casei* group obtained from NCBI GenBank, including *L. casei* ATCC 393^T^, *L. zeae* DSM 20178^T^, *L. chiayiensis* NCYUAS^T^, *L. rhamnosus* DSM 20021^T^, and *L. paracasei* JCM 8130^T^. FastANI was run using the default k-mer size of 16 and fragment length of 3000 bp. An ANI value of approximately 95–96% was used as the conventional species-level boundary.

### 2.2. Growth Characteristics, Acidification Kinetics, and H_2_O_2_ Production of L. casei

Overnight cultures of *L. casei* were inoculated into MRS broth at a final concentration of 0.1% (*v*/*v*) and incubated anaerobically at 37 °C. Bacterial growth was monitored by measuring the optical density at 600 nm (OD_600_) every 2 h using a microplate spectrophotometer (BioTek Instruments, Inc., Winooski, VT, USA). The acidification capability of the strain was evaluated by measuring the pH of the bacterial culture every 2 h throughout the incubation period. All measurements were performed in triplicate. To determine hydrogen peroxide (H_2_O_2_) production, the strain was streaked onto MRS agar supplemented with tetramethylbenzidine (TMB) and horseradish peroxidase (HRP) (Beijing Solarbio Science & Technology Co., Ltd., Beijing, China), followed by anaerobic incubation at 37 °C for 48–72 h. H_2_O_2_ production was assessed based on the TMB–HRP chromogenic reaction [11].

### 2.3. Analysis of Acid and Bile Salt Tolerance

*L. casei* single colonies from CaCO_3_-enriched MRS agar plates were inoculated into 5 mL of MRS broth and incubated at 37 °C for 24 h. Different pH gradients (2, 3, 4, 5, and 6) were applied to the culture medium, with pH 6 as the control. The final bile salt concentrations were also increased to 0% (control), 0.05%, 0.1%, 0.15%, and 0.2%. A bacterial suspension at 2% (*v*/*v*) was added to 5 mL of medium, and the mixture was incubated at 37 °C. Using a microplate reader, OD6_00_ values were measured at 0, 2, 4, 6, 8, 10, and 12 h. Three biological replicates were examined at each time point.

### 2.4. In Vitro Antimicrobial Assay

The agar well diffusion method was used to assess antibacterial activity. *Escherichia coli* (ATCC 25922), *Salmonella enterica* (ATCC 13076), *Staphylococcus aureus* (ATCC 29213), *Methicillin-resistant Staphylococcus aureus* (MRSA, ATCC 43300), *Salmonella enterica serovar Typhimurium* (ATCC 14028), and three EHEC isolates (DG-28, 54-G, and 6-G-11) isolated from diarrheic calves were used as indicator strains. Whole-genome sequencing identified the virulence genes *stx1A*, *hlyA*, *hlyB*, and *hlyD* in DG-28. Every bacterial strain was cultivated and standardized to a 0.5 McFarland turbidity. Mueller-Hinton (MH) agar plates were then evenly covered with 100 μL of bacterial solution. The untreated culture supernatant of *L. casei* was added to each well, and sterile MRS broth served as the negative control. Then, 100 μL aliquots were added to each well every 2 h during the 12-h incubation at 37 °C. Digital calipers were used to measure the diameters of the zone of inhibition. The same inhibition zone diameter criteria were applied to all indicator strains for comparative evaluation of antibacterial activity: <8 mm, negative (−); 8–12 mm, weak inhibition (+); 12–16 mm, moderate inhibition (++); and >16 mm, strong inhibition (+++) [12].

### 2.5. Antimicrobial Susceptibility Testing

Antimicrobial susceptibility testing was performed using the Kirby–Bauer disk diffusion method against 25 antimicrobial agents representing eight antibiotic classes, including β-lactams (ampicillin, amoxicillin, amoxicillin-clavulanic acid, ampicillin-sulbactam, piperacillin, piperacillin-tazobactam, cefotaxime, ceftazidime, cefepime, cefoxitin, ceftriaxone, and aztreonam), aminoglycosides (gentamicin, amikacin, streptomycin, and neomycin), fluoroquinolones (ciprofloxacin and levofloxacin), tetracycline, trimethoprim-sulfamethoxazole, chloramphenicol, florfenicol, azithromycin, polymyxin B, and fosfomycin.

Fresh overnight cultures were harvested by centrifugation, washed twice with sterile phosphate-buffered saline (PBS) to remove residual culture medium and acidic metabolites, and adjusted to a final concentration of 1 × 10^7^ CFU/mL. A 100 μL aliquot of the bacterial suspension was evenly spread onto MRS agar plates. Sterile antimicrobial disks were placed onto the agar surface using sterile forceps, and the plates were incubated under anaerobic conditions at 37 °C for 24 h. The diameters of the inhibition zones were measured using a Vernier caliper. Because standardized interpretive criteria for Lactobacillus spp. remain limited, the inhibition zone diameters were interpreted with reference to previously published criteria commonly adopted for lactic acid bacteria and were used for comparative evaluation of the antimicrobial susceptibility profile of the isolate rather than for clinical susceptibility determination [13,14].

### 2.6. Whole-Genome Sequencing and Bioinformatic Characterization of L. casei

Whole-genome bioinformatic analyses of *L. casei* were conducted after sequence acquisition. SPAdes v3.15.5 software was used to assemble raw sequencing reads, which were then analyzed for functional prediction and genomic annotation. Antibiotic resistance determinants were tested against the Comprehensive Antibiotic Resistance Database (CARD), and virulence-associated genes were predicted using VirulenceFinder v2.0.5. All analyses were conducted under standardized parameters to ensure comparability [15].

### 2.7. Hemolytic Activity, Autoaggregation, Coaggregation, and Hydrophobicity Assays

Freshly cultured isolates were placed on brain heart infusion (BHI) agar (Qingdao Hope Bio-Technology Co., Ltd., Qingdao, China) enriched with 5% (*v*/*v*) defibrinated sheep blood and incubated at 37 °C for 24 h to measure hemolytic activity. *S. aureus* ATCC 29213 was used as the positive control. Based on colony-associated clear zones, hemolytic patterns were categorized as α-, β-, or γ-hemolysis [16].

Bacterial cultures were revived from frozen stocks and subcultured overnight to obtain activated cultures. The activated cultures were then inoculated into MRS broth (2%, *v*/*v*) and incubated at 37 °C for 18 h before the autoaggregation assay. The cultures were centrifuged for 10 min at 8000 r/min, followed by three PBS washes and adjustment to an OD_600_ of 0.5. Following a 4-h incubation at 37 °C, 200 μL of the top layer was extracted for triplicate OD_600_ measurements. Autoaggregation (%) = [(A_0_ − A_t_)/A_0_] × 100.

For coaggregation analysis, suspensions of standard pathogenic strains were prepared using the same protocol. Equal volumes (1 mL each) of *L. casei* and pathogen suspensions were mixed, vortexed thoroughly, and allowed to stand. Following incubation at 37 °C for 2 h, 200 μL aliquots from the upper phase were collected for OD_600_ measurement. Coaggregation (%) = [(A_lp_ + A_pat_)/2 − A_lp+pat_]/[(A_lp_ + A_pat_)/2] × 100.

A 2 mL of the *L. casei* suspension and 0.4 mL of xylene or ethyl acetate, both purchased from Tianjin Zhiyuan Chemical Reagent Co., Ltd. (Tianjin, China), were combined, vortexed for 120 s, and then incubated at 37 °C for 1 h to perform a hydrophobicity analysis. The aqueous phase’s OD_600_ was measured. Hydrophobicity was calculated using the following formula: Hydrophobicity (%) = [(A_0_ − A_t_)/A_0_] × 100 [17].

Where A_0_ is the initial OD_600_ value, A_t_ is the OD_600_ value after incubation, A_lp_ is the initial OD_600_ value of the *Lacticaseibacillus casei* suspension, A_pat_ is the initial OD_600_ value of the pathogenic bacterial suspension, and A_lp+pat_ is the OD_600_ value of the mixed suspension of *Lacticaseibacillus casei* and the pathogenic bacterium after co-incubation.

### 2.8. Experimental Animals and Study Design

Male Kunming mice that were six weeks old were kept in specified pathogen-free (SPF) environments. All animal procedures were carried out in compliance with ARRIVE guidelines and approved by Xinjiang Agricultural University’s Ethics Committee (Approval No. 2024050). After a 1-week acclimatization, animals were randomly divided into four groups (*n* = 15 per group; total, *n* = 60). For 13 days, Group A (safety examination) was given an oral gavage of *L. casei* 2S-1 (200 μL, 1 × 10^8^ CFU/day). On day 13, Group B (preventive treatment) received an intraperitoneal injection of 200 μL of enterohemorrhagic *Escherichia coli* (EHEC) DG-28 (1 × 10^8^ CFU/mL) following the same probiotic pretreatment to establish an infection-associated intestinal inflammatory injury model. The inoculum concentration was confirmed by plate counting before administration. Group D (the blank control group) received equal volumes of sterile saline throughout the experiment. In contrast, Group C (the infection control group) only received the *E. coli* suspension on day 13. Every day, clinical indicators and body weight were tracked. Only tissues collected from mice euthanized at the experimental endpoint were used for histopathological, immunohistochemical, microbiome, and transcriptomic analyses. Tissues from animals that died during the experiment were excluded from these analyses. The major organs (heart, liver, spleen, lungs, kidneys, brain, stomach, testes, duodenum, jejunum, and colon) were removed, weighed, and organ-to-body weight indices were computed. For histopathological evaluation, four mice per group were analyzed by an investigator blinded to group allocation. Eleven sections from each mouse, representing the heart, liver, spleen, lung, kidney, brain, stomach, testis, duodenum, jejunum, and colon, were systematically examined across the entire tissue area at a fixed magnification. No predefined histopathological scoring system was applied; therefore, the findings were interpreted qualitatively. All tissues were fixed in 4% paraformaldehyde, paraffin-embedded, sectioned, and stained with hematoxylin and eosin. All reagents and consumables used for tissue fixation, paraffin embedding, sectioning, and H&E staining were purchased from Servicebio Technology Co., Ltd. (Wuhan, China). Body weight was recorded daily throughout the experiment. After euthanasia, major organs were collected and weighed for organ index calculation. Whole blood was analyzed using a BM830 automated hematology analyzer (Beijing Baolingman Sunshine Technology Co., Ltd., Beijing, China), and serum IL-6 levels were measured by enzyme-linked immunosorbent assay (ELISA). To assess bacterial translocation, aseptically collected liver, spleen, and blood samples were cultured on MRS agar plates at 37 °C for 24 h, and bacterial growth was subsequently examined.

### 2.9. Gut Microbiota Bioinformatics Analysis

Twelve duodenal content samples were collected for gut microbiota analysis. Three mice were randomly selected from each experimental group (*n* = 15), and each sample represented an independent biological replicate. The V4 region of the bacterial 16S rRNA gene was amplified using the forward primer 515F (5′-GTGCCAGCMGCCGCGGTAA-3′) and the reverse primer 806R (5′-GGACTACHVGGGTWTCTAAT-3′), paired-end (PE250) sequencing was carried out on the Illumina NovaSeq 6000 platform. Before library preparation and sequencing, PCR amplicons were verified, purified, and target fragments were retrieved. Raw reads were merged using FLASH (v1.2.11). Sequence denoising and ASV generation were performed using DADA2 within QIIME2, and taxonomic annotation was conducted against the SILVA 138.1 database. Beta diversity was assessed by PERMANOVA, and differential taxa were identified using LEfSe (v1.1.01) (LDA > 4.0). * (*p* < 0.05) and ** (*p* < 0.01) were the limits for statistical significance. Two-tailed tests were used for all statistical analyses [18,19].

### 2.10. Intestinal Immunohistochemistry

Samples of duodenal tissue that had been fixed in 4% PFA were sectioned, paraffin-embedded, and placed on microscope slides. Antigen retrieval was performed using sodium citrate buffer under heat-induced epitope retrieval conditions, following deparaffinization and rehydration. Endogenous peroxidase activity was blocked with 3% H_2_O_2_, and nonspecific protein binding was inhibited with 3% bovine serum albumin (BSA, Servicebio, GC305010) at room temperature. Tissue sections were incubated overnight at 4 °C with primary antibodies against mouse TNF-α (Affinity Biosciences Ltd., Changzhou, China; AF7014, 1:100), IL-1β (Wuhan Servicebio Technology Co., Ltd., Wuhan, China; GB11113, 1:100), Claudin-1 (Wuhan Servicebio Technology Co., Ltd., Wuhan, China; GB152543, 1:400), and ZO-1 (Wuhan Servicebio Technology Co., Ltd., Wuhan, China; GB115686, 1:1500). All primary antibodies were validated by the manufacturers for immunohistochemical detection in mouse tissues. After washing with PBS, the sections were incubated for 1 h at room temperature with an HRP-conjugated goat anti-rabbit IgG secondary antibody (Servicebio, 1:500). Chromogenic development was performed with a DAB detection kit, followed by hematoxylin counterstaining. Slides were then dehydrated, cleared, and mounted. A light microscope was used to view the stained slices. Positive staining intensity was quantified by integrated optical density (IOD) analysis using ImageJ 1.53. H-scores were calculated according to the following formula: H-score = (3 × High Positive%) + (2 × Positive%) + (1 × Low Positive%). Immunohistochemical analysis was performed in three mice per group. For each mouse, three independent duodenal sections and at least three non-overlapping microscopic fields per section were analyzed. Measurements from all sections and fields were averaged to obtain one value for each mouse, and each mouse was treated as one independent biological replicate for statistical analysis [20].

### 2.11. RNA Sequencing and Data Analysis

Total RNA was extracted from intestinal tissues collected from different experimental groups using TRIzol reagent (TaKaRa, Kyoto, Japan), and RNA-seq libraries were constructed [21]. Three biological replicates were included for each experimental group. Beijing Novogene Co., Ltd. (Beijing, China). performed high-throughput sequencing on an Illumina platform. After quality control, clean reads were aligned to the *Mus musculus* reference genome (Ensembl_100_mus_musculus_grcm38_toplevel) using HISAT2 (v2.0.5) with default parameters. Gene-level read counts were quantified using featureCounts (v1.5.0-p3) with default parameters. Reads with mapping quality scores below 10, improperly paired reads, and reads mapped to multiple genomic regions were excluded from gene-level quantification. Differential expression analysis was performed using DESeq2 (v1.20.0) based on raw read counts. Read counts were normalized using the DESeq normalization method, and differential expression was assessed using a negative binomial model. *p* values were adjusted for multiple testing using the Benjamini–Hochberg method. Genes with |log_2_(FoldChange)| ≥ 1 and an adjusted *p* value (padj) ≤ 0.05 were considered differentially expressed. GO and KEGG functional enrichment analyses of DEGs were performed using clusterProfiler (v3.8.1), with padj < 0.05 considered statistically significant [22].

### 2.12. Statistical Analysis

Statistical analyses were conducted using GraphPad Prism 8.0.2 software and ImageJ 1.53. Longitudinal body weight data were analyzed using a two-way repeated-measures ANOVA or a mixed-effects model (REML) when missing values were present. Survival curves were estimated using the Kaplan–Meier method and compared using the log-rank (Mantel–Cox) test. Differences between two groups were evaluated using an unpaired Student’s *t*-test, whereas comparisons involving three or more groups were performed by one-way ANOVA followed by Tukey’s post hoc multiple-comparison test. Statistical significance was defined as a two-sided *p* value < 0.05. For transcriptomic analysis, differentially expressed genes (DEGs) were identified using the thresholds of |log_2_FoldChange| ≥ 1 and an adjusted *p* value (adj. *p*) ≤ 0.05. Statistical significance is indicated as follows: *, *p* < 0.05. **, *p* < 0.01; ***, *p* < 0.001; and ns, not significant.

## 3. Results

### 3.1. Identification and Taxonomic Characterization of L. casei 2S-1

**Figure 1:**
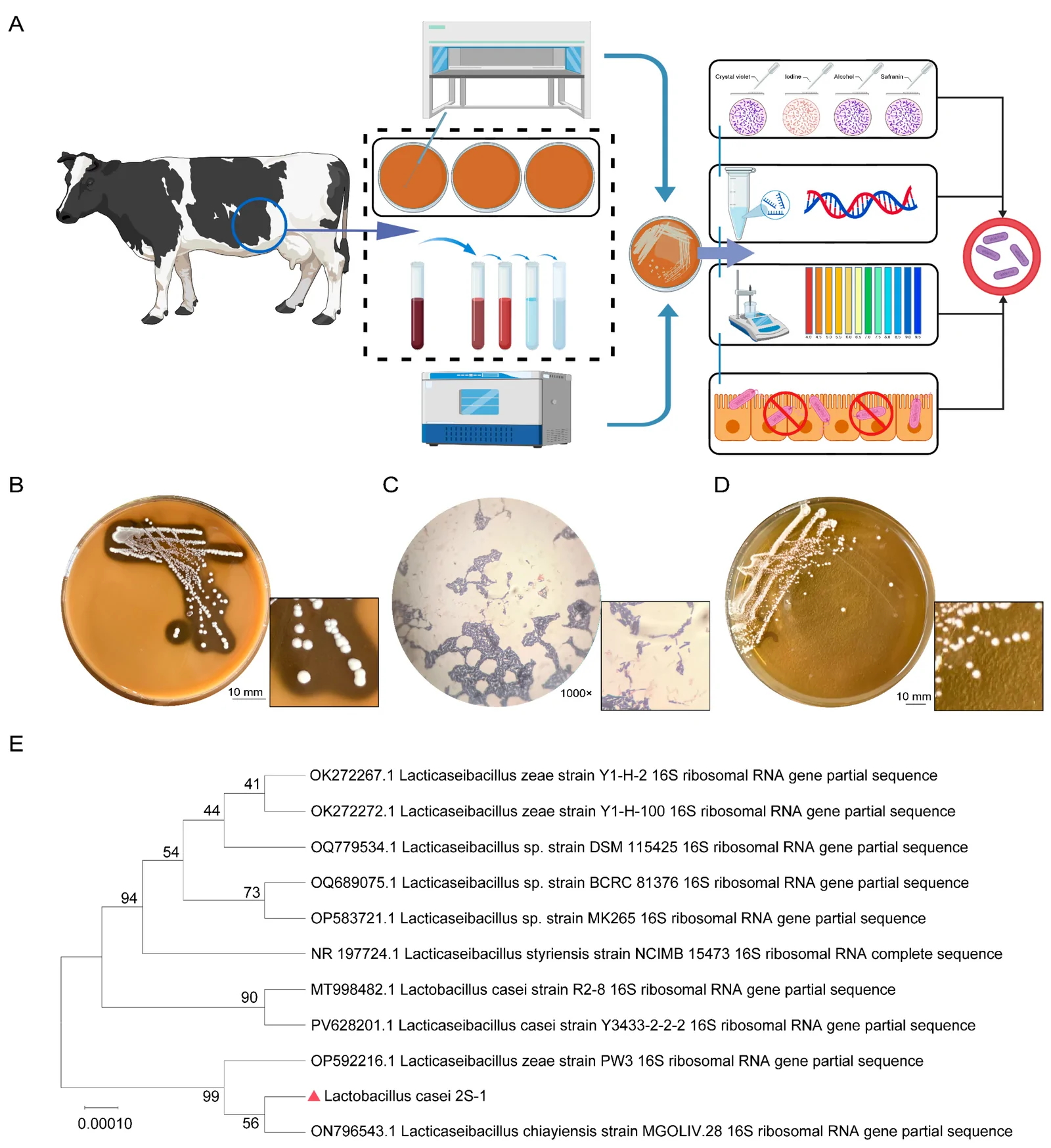
Isolation and identification of *L. casei*_2S-1. (**A**) Schematic illustration of the isolation and identification process of *L. casei* 2S-1; (**B**) Creamy-white smooth colonies on MRS medium and calcium-dissolution halo formation; (**C**) Gram-positive short rods morphology; (**D**) Absence of H_2_O_2_ production; (**E**) Phylogenetic tree based on *16S* rRNA gene sequences. Red triangles indicate the isolated lactic acid bacteria.

A bacterial strain was isolated from bovine rumen contents following the isolation and identification workflow shown in Figure 1A. After anaerobic cultivation on MRS agar at 37 °C for 24 h, the isolate produced smooth, moist, creamy-white circular colonies surrounded by calcium-dissolution halos (Figure 1B). Microscopic examination showed Gram-positive, short rod-shaped cells (Figure 1C), and no H_2_O_2_ production was detected (Figure 1D). Based on 16S rRNA gene sequencing and phylogenetic analysis (Figure 1E), the isolate was provisionally assigned to the *Lacticaseibacillus casei* group. Species-level identification was further supported by whole-genome ANI analysis. Strain 2S-1 showed 99.989% ANI with the *L. casei* type strain ATCC 393^T^, with a query-side alignment fraction of 99.41%. In contrast, ANI values with the type strains of *L. zeae*, *L. chiayiensis*, *L. rhamnosus*, and *L. paracasei* were 94.9354%, 88.7059%, 81.2859%, and 80.3817%, respectively, all below the conventional 95–96% species boundary. These results support the identification of strain 2S-1 as *Lacticaseibacillus casei*.

### 3.2. Probiotic Properties of L. casei 2S-1

**Figure 2:**
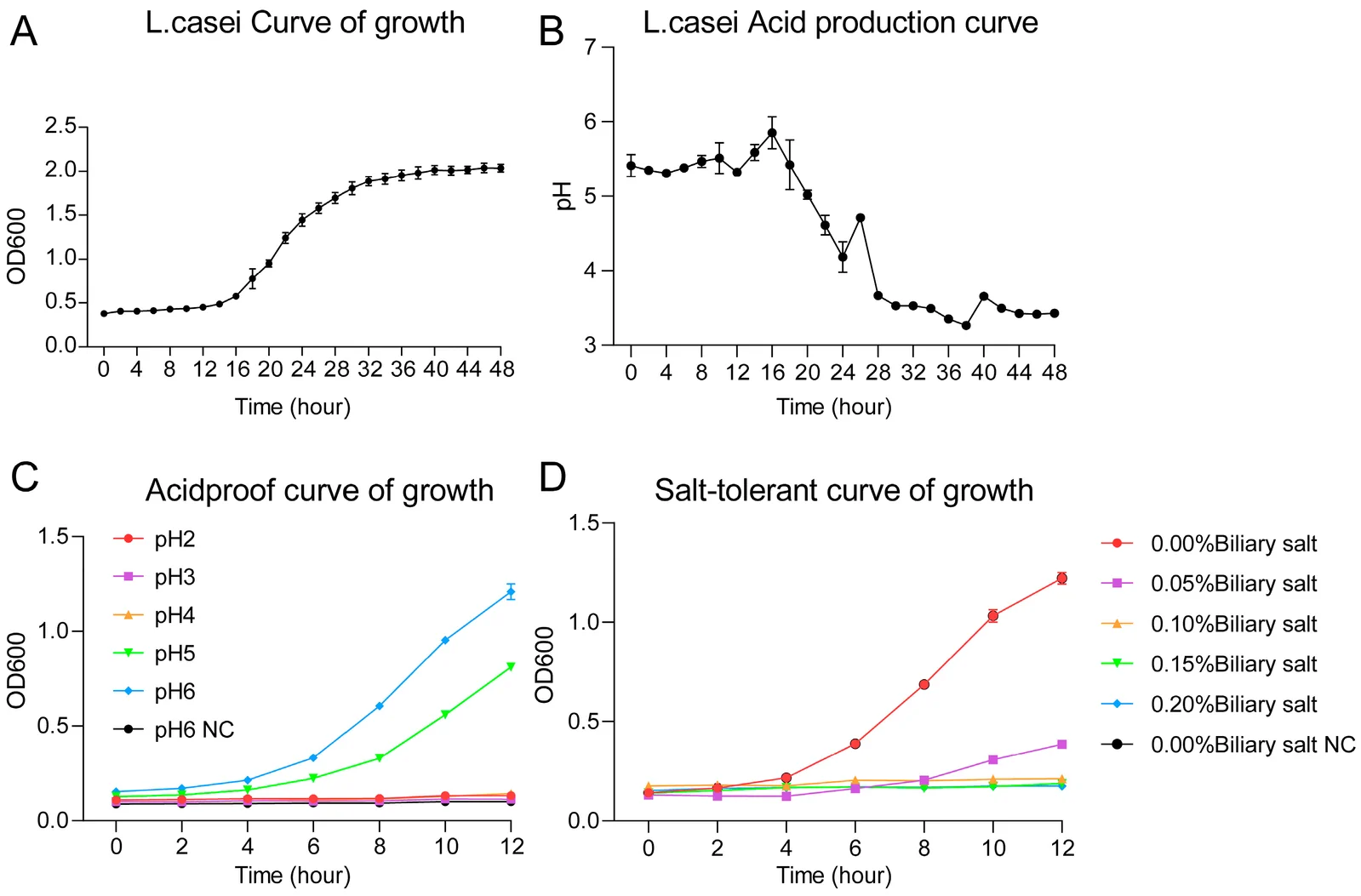
Growth characteristics of *L. casei*_2S-1. (**A**) Bacterial growth curve; (**B**) Acidification curve; (**C**) Acid tolerance growth curve; (**D**) Bile salt tolerance growth curve; NC, negative control.

The growth profile of *L. casei* 2S-1 followed a typical sigmoidal pattern, with a lag phase from 0–9 h, an exponential phase from 9–42 h, and a stationary phase initiated after 42 h of incubation (Figure 2A). During bacterial proliferation, the culture pH progressively declined and stabilized below pH 3.5 after approximately 30 h (Figure 2B). The strain showed increased OD_600_ values under mildly acidic conditions at pH 4–5, whereas no apparent increase in OD_600_ was observed at pH 2–3 (Figure 2C). Under bile salt stress, increased OD_600_ values were observed at bile salt concentrations of 0.05–0.10%, whereas only limited changes were detected at 0.15–0.20% (Figure 2D). These results indicate that *L. casei* 2S-1 possesses strong acidification capacity and tolerates moderately acidic conditions and low bile salt concentrations. In vitro, the untreated culture supernatant of *L. casei* 2S-1 showed antibacterial activity against three EHEC isolates from diarrheic calves and several other bacterial pathogens, including *Staphylococcus aureus*, *Escherichia coli*, and *Salmonella enterica* (Table 1).

### 3.3. In Vitro and In Vivo Safety Validation of L. casei_2S-1

**Figure 3:**
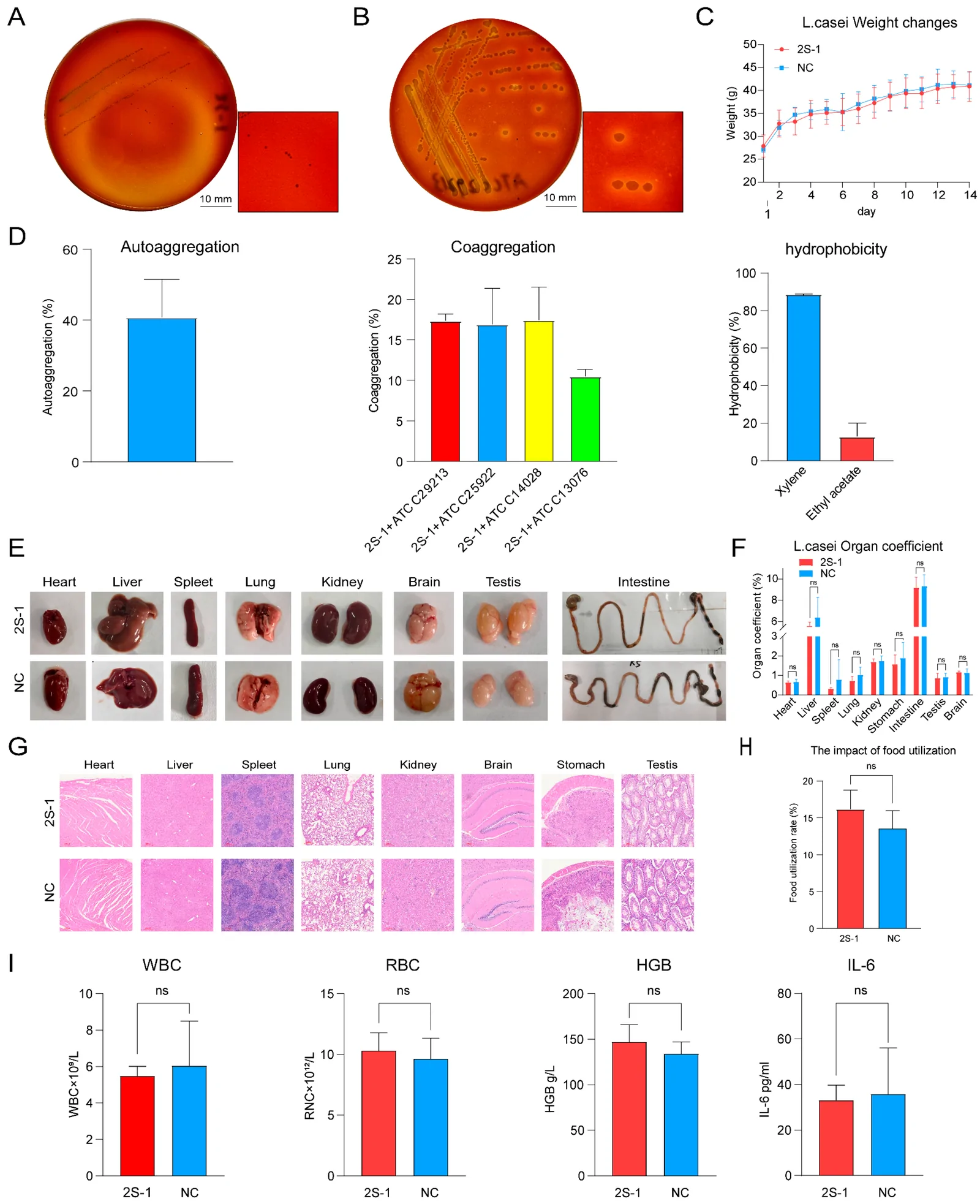
In vivo safety assessment of *L. casei*_2S-1. (**A**) Hemolytic activity of *L. casei* 2S-1 on sheep blood agar; a representative area with well-separated colonies is shown because acid production may locally alter the agar appearance in regions of dense growth; (**B**) Positive hemolysis control; (**C**) Changes in body weight in mice; (**D**) Autoaggregation, coaggregation, and hydrophobicity; (**E**) Gross appearance of major organs; (**F**) Organ indices; (**G**) H&E-stained histopathological sections; (**H**) Food utilization efficiency; (**I**) Hematological and inflammatory parameters. H&E, hematoxylin and eosin; WBC, white blood cell; RBC, red blood cell; HGB, hemoglobin; IL-6, interleukin-6; NC, negative control; ns, not significant.

**Figure 4:**
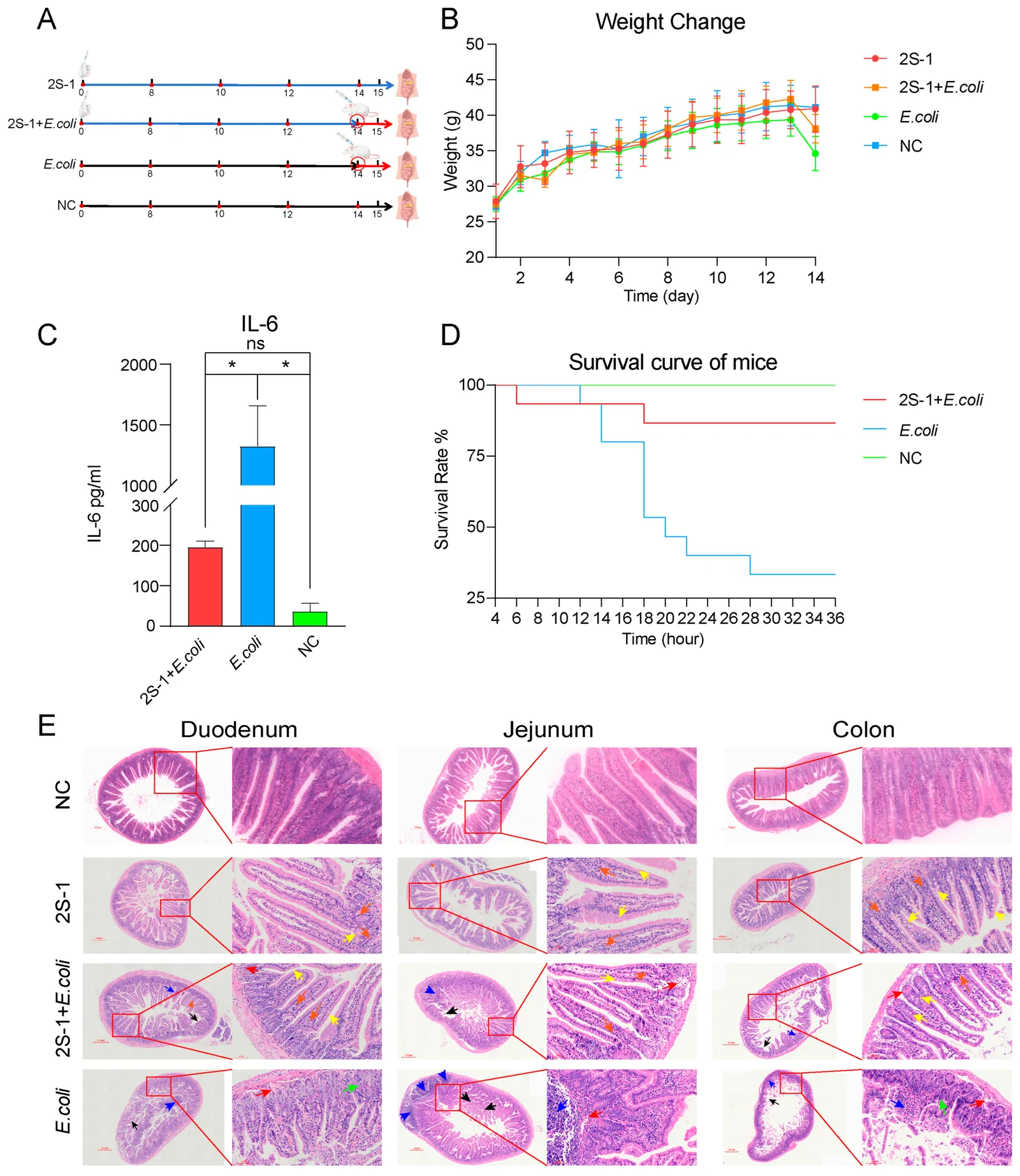
*L. casei*_2S-1 attenuates inflammation and extends survival in mice. (**A**) Experimental design flowchart; (**B**) 14-day body weight trajectories; (**C**) Serum pro-inflammatory cytokine IL-6 levels; (**D**) Kaplan–Meier survival curves; (**E**) Histopathological examination of the duodenum, jejunum, and colon showing villous destruction and crypt loss (black arrows), inflammatory cell infiltration (blue arrows), goblet cell depletion (green arrows), focal hemorrhage (red arrow), crypt structures (orange arrow) and goblet cells (yellow arrows). IL-6, interleukin-6; *E. coli*, *Escherichia coli*; NC, negative control; ns, not significant; *, *p* < 0.05.

No hemolytic activity was detected in *L. casei* 2S-1 (Figure 3A,B). In vivo safety assessment showed that body weight parameters did not differ from those of the control group (Figure 3C). The strain also showed positive autoaggregation, coaggregation, and cell surface hydrophobicity (Figure 3D). Organ indices remained within normal physiological ranges, and no gross pathological lesions were observed in major organs (Figure 3E–G and Figure 4E). Feed utilization efficiency was marginally increased (Figure 3H). Hematological parameters (WBCs, RBCs, hemoglobin concentrations, and serum pro-inflammatory cytokine levels) remained within normal ranges, without systemic inflammation or infection (Figure 3I). Moreover, no evidence of bacterial translocation was identified. Based on inhibition zone diameters (mm), the isolate was categorized as susceptible to ampicillin, amoxicillin-clavulanaic acid, cefepime, piperacillin, gentamicin, levofloxacin, tetracycline, chloramphenicol, azithromycin, ampicillin-sulbactam, piperacillin, amoxicillin and florfenicol; resistant to cefotaxime, aztreonam, trimethoprim-sulfamethoxazole, polymyxin B, cefoxitin, and fosfomycin; and intermediate susceptibility to the remaining antimicrobial agents. Whole-genome analysis identified no acquired antimicrobial resistance genes or virulence-associated genes based on the CARD and VirulenceFinder databases.

### 3.4. Preventive Efficacy of L. casei 2S-1 Against E. coli Infection in Mice

Mice received continuous oral administration of *L. casei* 2S-1 followed by intraperitoneal exposure to *E. coli* DG-28 according to the experimental design shown in Figure 4A. Mice in both the *E. coli* challenge group and the *L. casei* 2S-1-pretreated group displayed decreased body weight (Figure 4B). Serum IL-6 levels were higher in both groups; however, the IL-6 induction was significantly lower in the *L. casei* 2S-1 + *E. coli* group than in the *E. coli*-only group (Figure 4C). The final survival rate in the *E. coli* challenge group was less than 35%, and mortality started to show up 12 h after infection. On the other hand, mice pretreated with *L. casei* 2S-1 showed a marked improvement in survival, with a final survival rate of more than 85% (Figure 4D). *L. casei* 2S-1 pretreatment improved survival and attenuated infection-associated intestinal inflammatory injury following *E. coli* challenge. Histopathological examination of the duodenum in the *E. coli* challenge group revealed severe villous disruption, extensive inflammatory cell infiltration, and crypt deepening. In comparison, mice in the *L. casei* 2S-1 + *E. coli* group showed relatively intact villous organization, better-preserved mucosal architecture, and relatively preserved crypt morphology. In jejunal tissues, the *E. coli* group showed significant thinning of the intestinal wall, reduced villous density, disruption of microvillar continuity (black arrows), depletion of goblet cells (green arrows), and inflammatory cell infiltration (blue arrows). In the *L. casei* 2S-1 + *E. coli* group, the representative sections showed relatively preserved microvillar continuity, goblet cells, and villous architecture. In colonic tissues, the *E. coli* challenge group showed crypt loss, goblet cell depletion (black and green arrows), focal hemorrhagic lesions (red arrow), and intestinal wall thickening. Representative colonic sections from the *L. casei* 2S-1 + *E. coli* group showed relatively preserved crypt structures (orange arrow) and goblet cells (yellow arrow), with less apparent inflammatory infiltration, neutrophil accumulation, and intestinal wall thickening (Figure 4E).

### 3.5. Impact of L. casei_2S-1 on Mouse Gut Microbiota

**Figure 5:**
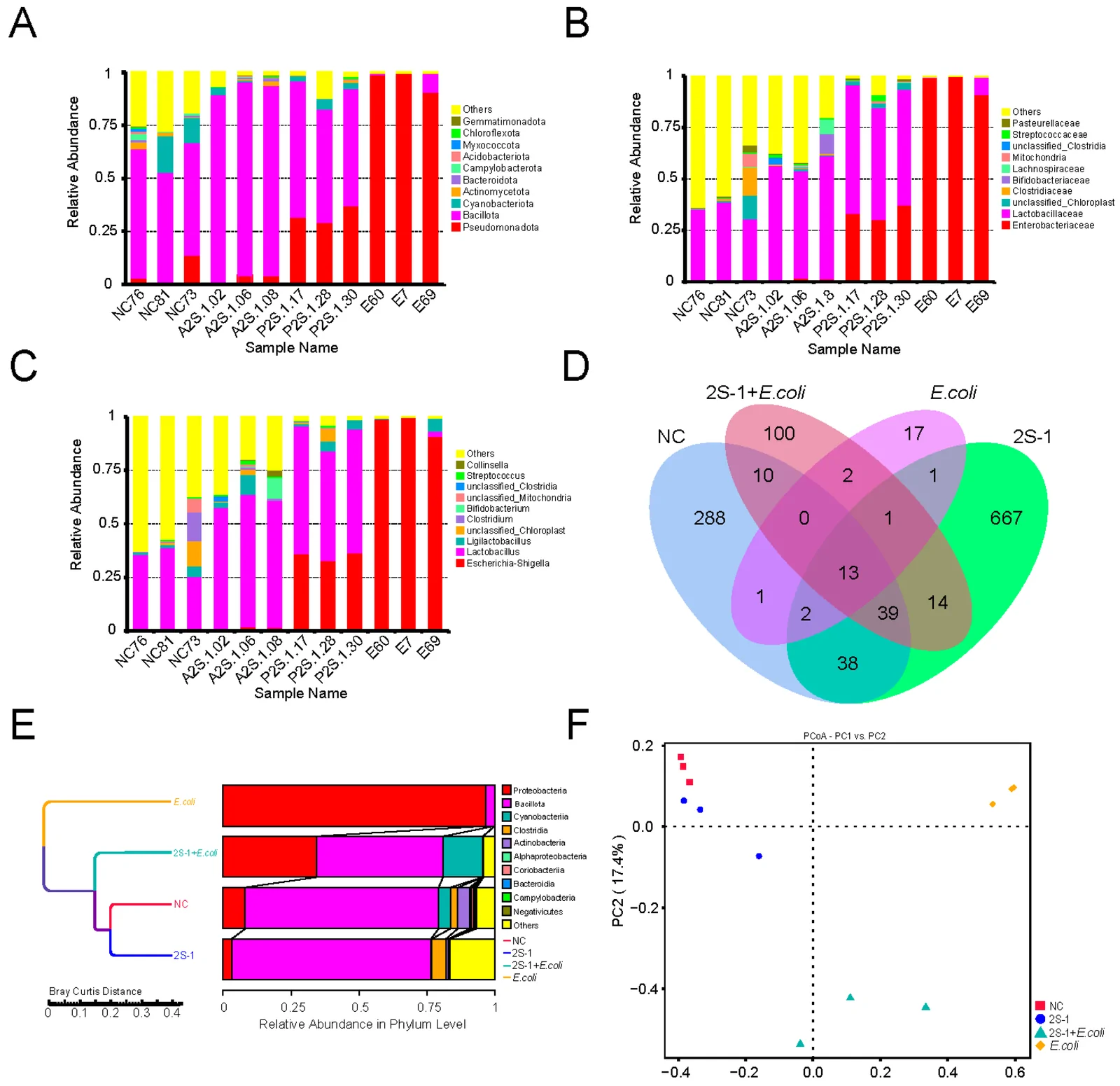
Effects of *L. casei* 2S-1 on gut microbial composition and community structure. (**A**) Phylum-level composition; (**B**) Family-level composition; (**C**) Genus-level composition; (**D**) Flower plot; (**E**) UPGMA clustering; and (**F**) Principal coordinate analysis (PCoA). UPGMA, unweighted pair-group method with arithmetic mean; PCoA, principal coordinate analysis; NC, negative control.

**Figure 6:**
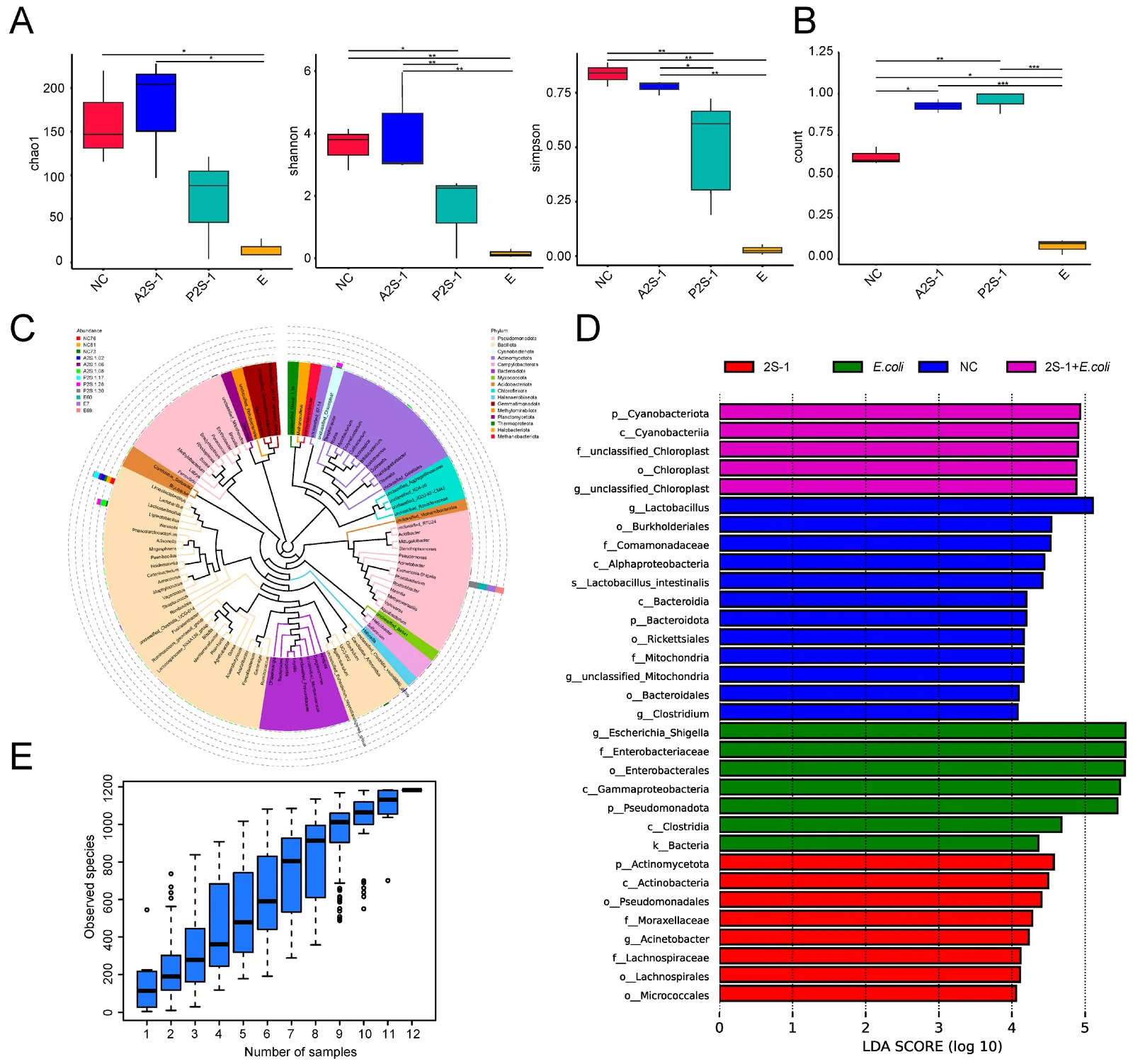
Effects of *L. casei* 2S-1 on microbial diversity and differential taxa. (**A**) Alpha-diversity indices; (**B**) Beta-diversity comparison; (**C**) Genus-level phylogenetic tree; (**D**) Linear discriminant analysis effect size (LEfSe); and (**E**) Species accumulation analysis. LEfSe, linear discriminant analysis effect size; LDA, linear discriminant analysis; NC, negative control; *, *p* < 0.05; **, *p* < 0.01; ***, *p* < 0.001.

*L. casei* 2S-1 pretreatment was associated with alterations in intestinal microbial composition and community structure. At the phylum level, the model group showed a disrupted Firmicutes ratio and a significant expansion of the pro-inflammatory phylum Proteobacteria. At the family and genus levels, the model group showed enrichment in potentially pathogenic taxa (i.e., Enterobacteriaceae) and depletion of beneficial bacterial genera such as Lactobacillus. However, the prevention group partially reversed these microbial changes (Figure 5A–C). The prevention group also showed higher OTU abundance in the flower plot (Figure 5D). UPGMA clustering and principal coordinate analysis (PCoA) revealed distinct differences in microbial community structure among groups (Figure 5E,F). Alpha- and beta-diversity analyses further showed partial restoration of microbial diversity after probiotic intervention (Figure 6A,B). The genus-level phylogenetic tree and LEfSe analysis identified distinct microbial taxa among the groups (Figure 6C,D), while species accumulation analysis supported these findings (Figure 6E). These results indicate that *L. casei* 2S-1 was associated with changes in gut microbial composition following EHEC infection.

### 3.6. Effects of L. casei_2S-1 on Intestinal Inflammatory Cytokines and Tight Junction Proteins

**Figure 7:**
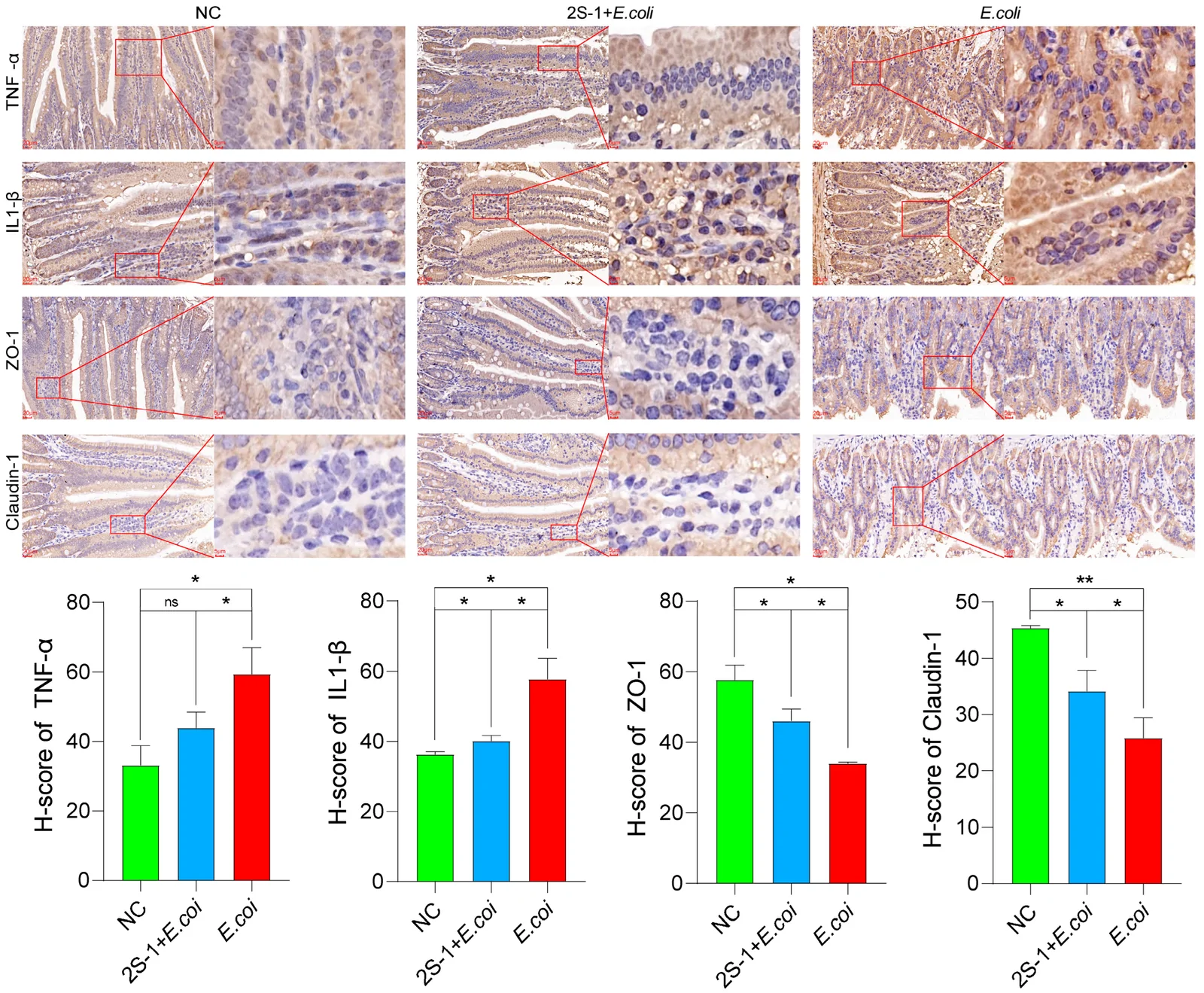
Immunohistochemical staining for TNF-α, IL-1β, ZO-1, and Claudin-1 in the mouse duodenum after *L. casei*_2S-1 intervention. TNF-α, tumor necrosis factor alpha; IL-1β, interleukin-1 beta; ZO-1, zonula occludens-1; NC, negative control; *, *p* < 0.05; **, *p* < 0.01; ns, not significant.

*L. casei* 2S-1 effectively protected against *E. coli* infection-induced inflammatory responses and disruption of tight junction proteins. Duodenal tissues from the *E. coli* challenge group had more immunoreactive cells and higher levels of the pro-inflammatory cytokines TNF-α and IL-1β than those from the control and preventive groups, as determined by immunohistochemistry (*p* < 0.05). TNF-α and IL-1β expression were significantly lower in the preventive group than in the *E. coli* group (*p* < 0.05), suggesting attenuation of local intestinal inflammation, and the preventive group had significantly fewer immunoreactive cells. The EHEC group showed lower immunostaining intensity for ZO-1 and Claudin-1 than the control and prevention groups (*p* < 0.05). Representative sections also showed a less continuous staining pattern along the intestinal epithelium. In the prevention group, the immunostaining intensity was higher and the epithelial staining pattern appeared more continuous than in the EHEC group (Figure 7).

### 3.7. Intestinal Transcriptomic Analysis of L. casei_2S-1 in E. coli-Induced Murine Enteritis

**Figure 8:**
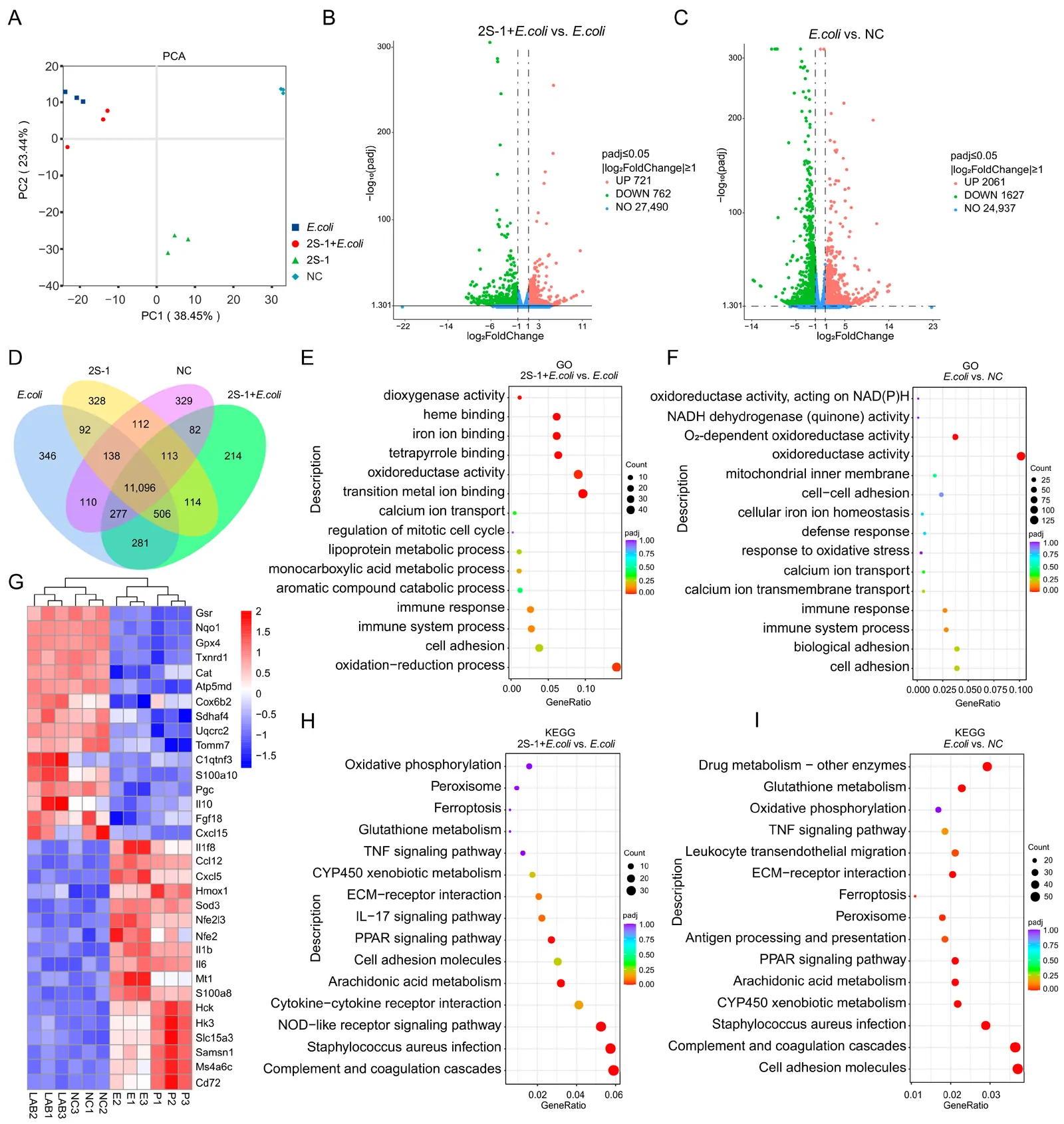
Intestinal transcriptomic analysis of *L. casei*_2S-1 in *E. coli*-induced murine enteritis. (**A**) PCA plot; (**B**) Volcano plot (prevention vs. *E. coli* group); (**C**) Volcano plot (*E. coli* group vs. NC); (**D**) Venn diagram of DEGs; (**E**) GO enrichment (prevention vs. *E. coli* group); (**F**) GO enrichment (*E. coli* group vs. NC); (**G**) Clustering heatmap of DEGs; (**H**) KEGG enrichment (prevention vs. *E. coli* group); (**I**) KEGG enrichment (*E. coli* group vs. NC). PCA, principal component analysis; DEGs, differentially expressed genes; GO, Gene Ontology; KEGG, Kyoto Encyclopedia of Genes and Genomes; NC, negative control; *E. coli*, *Escherichia coli*.

Transcriptomic profiling of intestinal tissues was conducted to characterize global gene expression changes associated with *E. coli* infection and probiotic intervention. PCA showed clear clustering among all groups (Figure 8A). Differential gene expression analysis comparing the prevention and model groups identified 1483 significantly dysregulated genes (Figure 8B), whereas comparison of the model and control groups revealed 3688 significantly dysregulated genes (adjusted *p* ≤ 0.05, |log_2_FC| ≥ 1; Figure 8C). The Venn diagram (Figure 8D) illustrates shared and unique sets of DEGs across the four groups. GO enrichment analyses comparing the prevention and model groups identified differential enrichment of pathways associated with the “oxidation-reduction process” and “oxidoreductase activity” (Figure 8E). GO enrichment analyses comparing the model and control groups showed significant enrichment of biological processes associated with oxidative stress and mitochondrial function (FDR < 0.05; Figure 8F). Hierarchical clustering heatmaps revealed marked upregulation of canonical pro-inflammatory genes (*IL-1β* and *IL-6*), confirming successful development of the inflammatory enteritis model. EHEC infection downregulated genes associated with mitochondrial function and oxidative metabolism (*Uqcrc2*, *Nqo1*, and *Atp5md*), while upregulating genes (*Hmox1*, *Sod3*, and *Nfe2l3*), suggesting transcriptional changes associated with oxidative stress and mitochondrial function. Compared with the other experimental groups, the safety assessment group showed higher expression levels of *S100a10* and *C1qtnf3* (Figure 8G). The prevention group showed attenuated enrichment of oxidative stress- and mitochondrial function-related pathways, suggesting transcriptional changes associated with redox regulation after probiotic intervention (Figure 8H). KEGG pathway analyses further supported these observations. Comparisons between the model and control groups showed significant enrichment not only of classical inflammatory pathways (i.e., cell adhesion molecule signaling) but also of pathways associated with oxidative stress and mitochondrial dysfunction (i.e., “PPAR signaling pathway,” “cytochrome P450-mediated xenobiotic metabolism,” “oxidative phosphorylation,” and mitochondrial dysfunction-related signaling pathways). These findings indicate transcriptional alterations associated with mitochondrial function and oxidative stress following EHEC infection (Figure 8I).

## 4. Discussion

Antimicrobial resistance has become one of the most critical challenges of global public health systems. Recent predictive models found that antimicrobial resistance could account for approximately 10 million deaths annually worldwide by 2050. In modern animal production systems, the irrational and excessive use of antibiotics remains widespread, resulting in a severe crisis of antimicrobial resistance. This phenomenon not only compromises animal health and limits the sustainable development of the livestock industry, but also promotes the accumulation and dissemination of resistance factors through the food chain, posing substantial threats to public health and human welfare [23,24]. This work isolated a novel strain of *Lacticaseibacillus casei* (*L. casei* 2S-1) from the rumen of cattle in Xinjiang. Three benefits were demonstrated by this strain. Firstly, whole-genome analysis did not identify acquired antimicrobial resistance genes using the CARD. However, the isolate exhibited phenotypic resistance to several antimicrobial agents, including cefotaxime, aztreonam, polymyxin B, and fosfomycin. This discrepancy may reflect resistance mechanisms or genetic variants that are not currently represented or sufficiently characterized in the database, as well as other factors such as altered gene expression, membrane permeability, or efflux activity. CARD enables the identification of antimicrobial resistance determinants based on both sequence homology and resistance-associated mutations; therefore, the discrepancy between the genomic prediction and phenotypic susceptibility profile should not be attributed to an inability of CARD to detect intrinsic or mutation-associated resistance. Therefore, genomic predictions and phenotypic antimicrobial susceptibility results should be interpreted together when evaluating the safety profile of probiotic candidates. Secondly, the strain demonstrated a variety of probiotic traits, including immunoregulatory properties, growth-promoting capabilities, and antibacterial activity against pathogens. Lastly, the strain showed tolerance to acidic and bile salt conditions. Collectively, these findings support the prophylactic potential of *L. casei* 2S-1 against infection-associated intestinal inflammation. Nevertheless, additional studies are required to further evaluate its safety, efficacy, and application potential before practical use.

**Figure 9:**
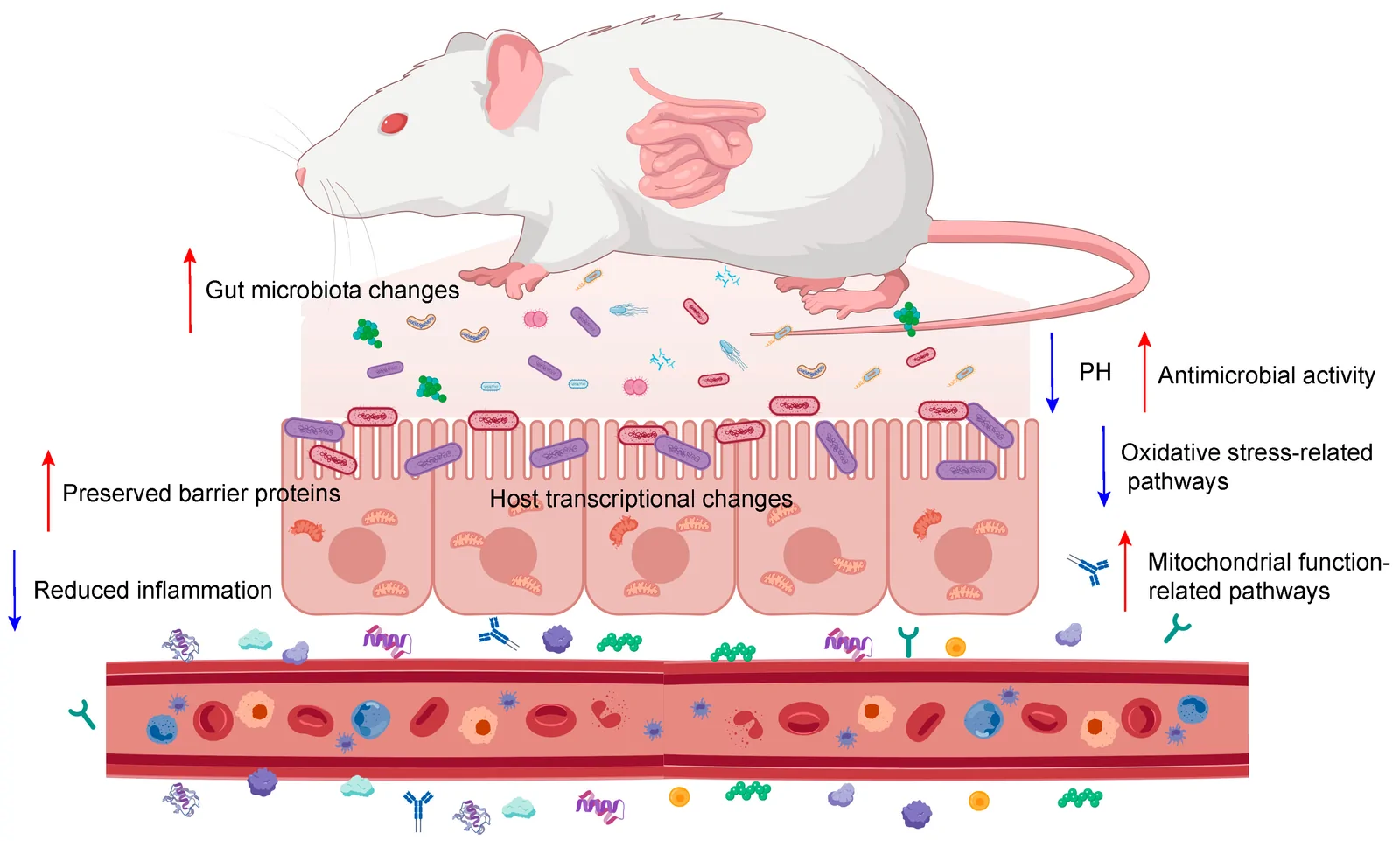
Proposed working model illustrating the potential protective effects of *L. casei* 2S-1 against infection-associated intestinal inflammation.

Figure 9 presents a proposed working model summarizing the changes associated with the preventive effects of *L. casei* 2S-1 against infection-associated intestinal inflammation. The maintenance of intestinal microecological homeostasis in livestock is dependent on diverse populations of beneficial microorganisms, among which lactic acid bacteria represent pivotal keystone taxa involved in preserving intestinal physiological stability and promoting host health [25]. In this study, *L. casei* 2S-1 was isolated and identified from the rumen of cattle in Xinjiang and demonstrated probiotic potential. Compared with strains isolated from conventional fermented food sources [26], this ruminant-derived isolate had favorable environmental adaptability and functional performance. The strain reduced the pH of the culture medium, and its untreated culture supernatant exhibited antimicrobial activity against several intestinal pathogens. This finding aligns with accepted probiotic selection standards that emphasize the antibacterial properties of organic acids produced by lactic acid bacteria [27]. Furthermore, *L. casei* 2S-1 showed limited growth at 0.2% bile salts based on OD_600_ measurements. Because only in vitro growth under bile salt stress was evaluated, additional studies using simulated gastrointestinal conditions or viability assays are warranted to further characterize its gastrointestinal tolerance. Lastly, the lack of hemolytic activity was verified through in vitro safety testing, supporting its preliminary safety profile and providing a basis for its further evaluation as a potential functional feed additive.

Competitive exclusion, including competition for nutrients and adhesion sites, has been proposed as a potential mechanism by which probiotics may inhibit pathogenic bacteria [28,29,30,31]. However, pathogen colonization and competitive exclusion were not directly evaluated in the present study. Prior research has demonstrated that when piglets are exposed to enterotoxigenic *E. coli* (ETEC), their immune systems are suppressed and the pro-inflammatory cytokine IL-6 is significantly upregulated. However, pretreatment with microencapsulated probiotics reduced serum IL-6 levels in ETEC-challenged piglets [32]. Consistent with these results, this study showed that *L. casei* intervention significantly reduced IL-6 production. Although *E. coli* infection increased IL-6 expression, *L. casei* pretreatment was associated with a lower IL-6 level (*p* < 0.001), suggesting a potential association with reduced IL-6-mediated inflammatory responses. *L. casei* 2S-1 was associated with increased microbial diversity and altered gut microbial composition following EHEC infection. However, additional research using a combination of metabolomic analysis and host immunological profiling to examine the underlying molecular pathways is necessary to determine whether this probiotic strain directly affects tissue inflammatory responses.

Immunohistochemical analyses conducted in this study revealed that duodenal tissues from EHEC-induced enteritis mice showed significantly upregulated pro-inflammatory mediators (TNF-α and IL-1β). The immunostaining intensity of the tight junction proteins Claudin-1 and ZO-1 was reduced, and their epithelial staining patterns appeared less continuous. These results are consistent with the current understanding in the field [33,34], which indicates that excessive production of inflammatory mediators, particularly TNF-α, directly disrupts the integrity of the intestinal epithelial barrier. Upregulated TNF-α and IL-1β levels may exacerbate local inflammation and promote epithelial tissue injury, while reduced immunostaining intensity and less continuous staining patterns of Claudin-1 and ZO-1 are associated with impaired intestinal mucosal barrier integrity.

In this study, integrated transcriptomic analyses, including volcano plot visualization, Venn diagram analysis, hierarchical clustering heatmaps, and functional enrichment analyses based on Gene Ontology (GO) and Kyoto Encyclopedia of Genes and Genomes (KEGG) pathways, were used to characterize the molecular landscape of EHEC-induced murine enteritis and the host transcriptional changes associated with probiotic pretreatment. Volcano plot analyses showed substantial changes in gene expression profiles following EHEC infection. Venn diagram analyses revealed alterations in the expression of inflammation-associated genes following probiotic pretreatment. Hierarchical clustering heatmaps further demonstrated distinct transcriptional profiles among the experimental groups. They validated the successful establishment of the inflammatory model and demonstrated differences in global transcriptional profiles among the experimental groups [35,36]. Functional enrichment analyses further linked the observed phenotypes to enriched biological pathways. Pathways associated with oxidative stress, mitochondrial function, and cytokine-mediated inflammatory signaling were enriched following EHEC infection. In comparison, probiotic pretreatment was associated with altered enrichment of these pathways, suggesting transcriptional changes related to oxidative stress and inflammatory responses. These transcriptomic findings indicate that probiotic intervention was associated with alterations in host transcriptional profiles and enriched biological pathways during EHEC-induced enteritis.

## 5. Conclusions

In conclusion, prophylactic administration of *Lacticaseibacillus casei* 2S-1 improved survival and attenuated infection-associated intestinal inflammatory injury in mice challenged with EHEC DG-28. These effects were accompanied by reduced inflammatory responses, relatively preserved Claudin-1 and ZO-1 immunostaining, alterations in gut microbial composition, and host transcriptional changes associated with oxidative stress and mitochondrial function. In vitro analyses further supported the probiotic potential and preliminary safety profile of this strain. Collectively, these findings identify *L. casei* 2S-1 as a promising candidate for the prevention of infection-associated intestinal inflammation. Nevertheless, additional studies using direct functional assays and independent animal models are required to confirm the underlying mechanisms, long-term safety, and practical applicability of this strain.

## Figures and Tables

**Table 1:** In vitro antibacterial activity of the untreated culture supernatant of *L. casei* 2S-1 against indicator strains.

Strains	Type	Antibacterial Circle Diameter (mm)	Decision Outcomes
ATCC 29213	*Staphylococcus aureus*	11.55	+
ATCC 25922	*Escherichia coli*	12.73	++
ATCC 14028	*Salmonella enterica serovar Typhimurium*	12.34	++
ATCC 13076	*Salmonella*	11.84	+
ATCC 43300	MRSA	13.19	++
DG-28	EHEC	13.07	++
54-G	EHEC	14.29	++
6-G-11	EHEC	12.94	++

Antibacterial activity was classified according to inhibition zone diameter as follows: 8–12 mm, weak inhibition (+); 12–16 mm, moderate inhibition (++).

## Data Availability

The whole-genome sequence of *L. casei* 2S-1 has been deposited in GenBank under accession number JBXGQF000000000.1. The raw 16S rRNA gene sequencing reads generated in this study have been deposited in the NCBI Sequence Read Archive under BioProject accession number PRJNA1474799. The raw transcriptome sequencing reads generated in this study have been deposited in the NCBI Sequence Read Archive (SRA) database under BioProject accession number PRJNA1474726.
